# Influence of bilateral transcranial direct-current stimulation on muscle strength and respiratory endurance: Randomized, placebo-controlled, double-blind trial protocol

**DOI:** 10.1016/j.mex.2024.102939

**Published:** 2024-09-18

**Authors:** Elder Nascimento Pereira, Fernando Zanela da Silva Arêas, Elizângela Kuster, Márcia de Lorena Serra Maia, Jessica Oliveira da Silva, Laila Ramires Norbim, Jorge Henriques, Guilherme Peixoto Tinoco Arêas

**Affiliations:** a PhD student in Biosciences, specialization branch in Neurosciences, University of Coimbra, Portugal; b Center for Health Sciences, Physical Therapy, Federal University of Espírito Santo, Vitória, ES, Brazil; c Laboratory of Neurorehabilitation and Neuromodulation, Department of Physiological Sciences, Federal University of Espírito Santo, Vitória, ES, Brazil; d Laboratory of Human Physiology, Department of Physiological Sciences, Federal University of Amazonas, Manaus, AM, Brazil; e Department of Informatics Engineering, Center for Informatics and Systems of the University of Coimbra, Coimbra, Portugal; f Advisor in the PhD program in Biosciences, Department of Life Sciences, University of Coimbra, Coimbra, Portugal

**Keywords:** Motor cortex, Bilateral transcranial stimulation, Respiratory muscle strength, Transcranial direct current stimulation

## Abstract

Transcranial direct current stimulation (tDCS) has become established as an effective therapeutic approach, employed to modulate cortical excitability in various conditions. Nonetheless, few studies have assessed the use of tDCS in improving respiratory performance both in healthy and in subjects with respiratory disfunction. This randomized double-blind placebo-controlled trial evaluated the outcomes of lung function, strength of inspiratory muscles, general strength after intervention with bilateral tDCS both in young and elderly female subjects. Eighty subjects were randomized into four groups divided by age (40 young and 40 elderly) and intervention vs. placebo. After a basal (day 1) evaluation all subjects performed two evaluation/intervention rounds with 48 to 72 h interval. Lung function evaluated with spirometry evaluation with Forced vital capacity (FVC), Forced Expiratory Volume in 1 S (FEV_1_), FEV_1_/FVC Ratio, Maximal Voluntary Ventilation (MVV); Dynamic Inspiratory muscle strength evaluated with Powerbreathe and general strength with dynamometer. This study intends to understand the behavior of respiratory muscle strength and endurance after intervention with bilateral cathodal tDCS over the primary motor cortex in healthy young and elderly subjects, as a bridge for larger studies both in healthy and rehabilitation setting.

## Graphical abstract

**Figure:**
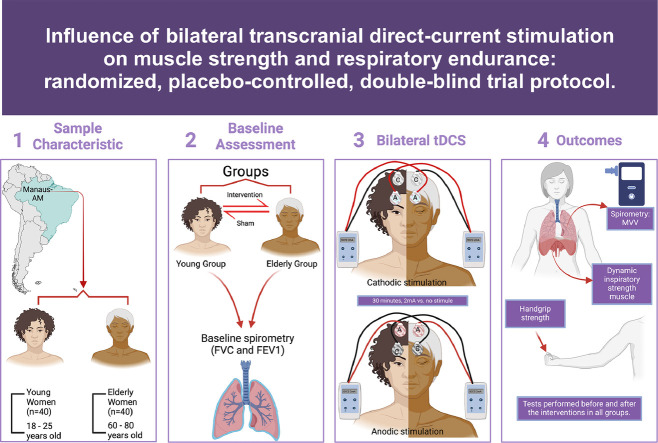

Specifications table

**Table:** 

Subject area:	Neuroscience
More specific subject area:	Effects of bilateral cathodal tDCS application in muscle strength and pulmonary performance in the young and elderly population.
Name of your protocol:	Bilateral transcranial direct-current stimulation on muscle strength and respiratory endurance: randomized, placebo-controlled, double-blind trial protocol
Reagents/tools:	Bilateral tDCS: Neuromodulator from Microestim Foco Research, NKL, Santa Catarina, Brazil. Forced vital capacity (FVC), Forced Expiratory Volume in 1 S (FEV1), FEV1/FVC Ratio, Maximal Voluntary Ventilation (MVV): SPM-A spirometer CONTEC™, China Powerbreathe K5 equipment (Powerbreathe, Southam, England): S-INDEX, Flow and Volume. Handgrip strength: hydraulic dynamometer (Saehan, Beijing, China).
Experimental design:	Randomized placebo-controlled double-blind trial. Four groups (*n* = 68), test (young and elderly, *n* = 34) and placebo (young and elderly, *n* = 34). Outcome measures were evaluation of lung function, strength of inspiratory muscles, general strength. After a basal evaluation, the outcomes were tested in two separated moments before and after bilateral cathodal tDCS stimulation both comparing in the same age group and comparing elderly versus young.
Trial registration:	U1111–1310–2605, International Clinical Trial Registry Platform
Ethics:	The study was conducted in line with recommendations from the Declaration of Helsinki and received ethical approval from the Ethics Committee of the Federal University of the Amazonas, Brazil [CAAE66350622.0.0000.520, evaluation 315.763].
Value of the Protocol:	tDCS is proven to be a valuable tool in rehabilitation and performance enhancement in several areas, but with little or no work done in respiratory function or respiratory rehabilitation. The main objective is to evaluate the expected improvement of the respiratory muscle strength and resistance after intervention with bilateral tDCS over the primary motor cortex in healthy young and elderly subjects. It is a randomized, double-blinded study designed to study the possibility of using tDCS for enhance respiratory performance in healthy subjects or in rehabilitation programs after respiratory injury. We highlight the construction of the study, allowing the comparison between the response in the young and the elderly.

## Background

Transcranial direct current stimulation (tDCS) has become established as an effective therapeutic approach, employed to modulate cortical excitability in various conditions, including depression or post-stroke rehabilitation. It involves the placement of two or more electrodes on the scalp, delivering a 1–2 mA current to a specific cortical region [1]. The rapid effects of low intensity neurostimulation alters action potential leading to depolarization or hyperpolarization [2,3]. While tDCS, particularly applied to the primary motor cortex (M1), has prompted investigation into its potential for improving motor function in both healthy subjects and various conditions with the expectation that it facilitates neuroplasticity and brain functional/structural reorganization. This is achieved through the modification of synaptic bindings by inductive long-term potentiation and long-term depression, as well as the modulation of neuronal resting membrane potential [[4], [5], [6]]. The assembly of the tDCS can be done unilaterally or bilaterally, and with anodic or cathodic stimulation. Interestingly, cathodal stimulation can have different effects depending on the current intensity. Studies have shown that 2.0 mA has an excitatory effect, while intensities of 1.0 mA and 3.0 mA seem to cause inhibition of the cerebral cortex [[7], [8], [9], [10], [11]]. A recent meta-analysis showed bilateral tDCS was more effective to improve motor performance in healthy individuals, while unilateral tDCS was stronger in rehabilitation of pathological conditions such as stroke [12].

Voluntary control of breathing involves the M1 cortex and premotor region, adding a major cortical component activated during exercise, speech, or voluntary respiratory maneuvers [[13], [14], [15]]. Nevertheless, other mechanisms participate in this control, such as pH or pCO_2_ [16]. Few studies have assessed the use of tDCS in improving respiratory performance both in healthy and in subjects with respiratory disfunction. De Carvalho et al., [17] evaluated two patients with spinal cord injury who were having difficulty weaning off tracheostomy. The authors demonstrated a slight increase in respiratory muscle strength in both patients. Andrade et al., [18] performed unilateral tDCS targeting the motor area of the diaphragm on the left side in patients in the intensive care unit with COVID-19. They showed that patients who received stimulation combined with physiotherapy had a shorter length of stay. However, no assessment of respiratory function values was conducted. On the other hand, Azabou et al., [16] demonstrated that unilateral tDCS can decrease the excitability of the corticospinal pathway of the ventilatory motor system, particularly the diaphragmatic function. Despite some studies failing to confirm the real impact of tDCS on respiratory function, others demonstrate decreased corticospinal excitability over the motor respiratory pathway, increased muscle consistency, and heightened activity when applied to the spinal motor area. However, to date, no study has assessed the effects of bilateral tDCS in motor areas and the impacts on the strength and resistance of respiratory muscles

### Research questions

- Compared to controls, is cathodic bilateral tDCS stimulation effective in improving muscle strength and respiratory performance?
- Will there be a change in the effects of cathodic bilateral tDCS when performed in the young vs. elderly regarding muscle strength gain/better respiratory performance?

## Description of protocol

Methods details

### Ethical approval

The study was conducted in line with recommendations from the Declaration of Helsinki and received ethical approval from the IRB of the Federal University of the Amazonas, Brazil [CAAE66350622.0.0000.520].

### Sample size and subject recruitment

The volunteers were recruited after posting invitation posters on the campus of the Federal University of Amazonas. No compensation was offered for participation in the experiment. All the subjects were female. The rationale for this was the ease of recruiting female participants, since there are activities taking place on the university campus only for elderly female.

Were inclusion criteria for the young group – ages comprehended between 18 and 25, no medical history of cardiovascular, pulmonary or neurologic conditions affecting the respiratory tract. Regarding the elderly group the inclusion criteria were the same, except the timeframe, considering ages between 60 and 80 years of age. Exclusion criteria were subjects out of the timeframe requested by the study protocol in both groups, medical history of cardiovascular, pulmonary or neurologic conditions. Also, in the selection of volunteers for the young group, those who were at the time of their menstrual period were excluded to avoid any hormonal influence on the results.

For the sample size, inspiratory muscle strength will be used through the dynamic inspiratory pressure (*S index*). As there is no study that evaluated this type of variable associated with tDCS, we will accept a priori a Cohen's size effect *F* = 0.25 (Small Effects). For sample size, ANOVA two-way repeated measures (Between-within) was calculated. Parameters used were size effect 0.25, α = 0.05, β = 80 %, 2 groups, 2 measurements (tDCS stimuli and Sham stimuli), correction between measurements = 0.5 and correction of no-sphericity ε = 1. Accepting a possible 20 % of sample loss, a sample calculation of 40 volunteers was selected both for the elderly and young group, with a global total of 80 subjects and G power 3.1 (University of Dusseldorf, Dusseldorf, Germany) was used for calculation.

**Fig. 1:**
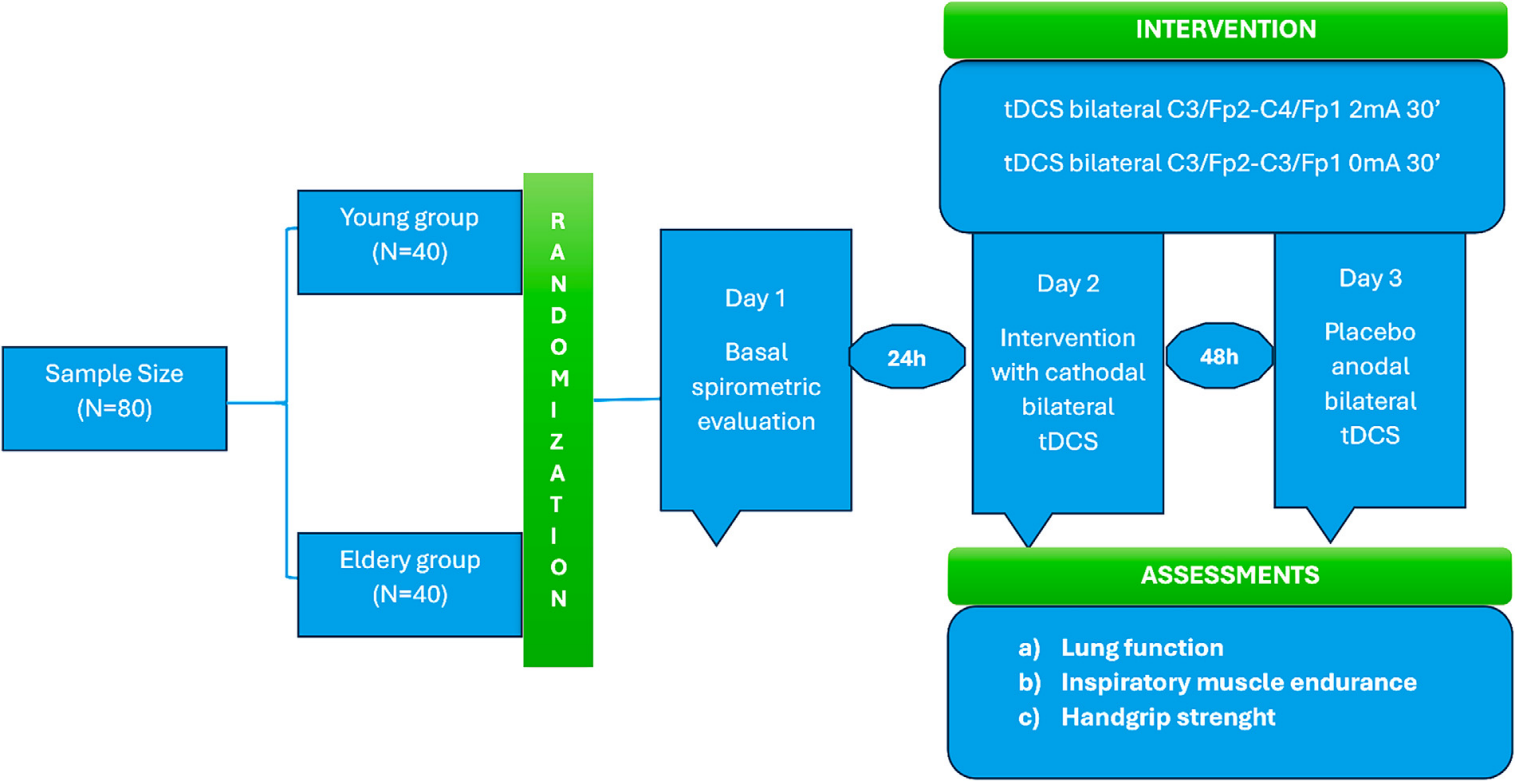
Study flowchart.

The stratification randomization (age, BMI, FVE1 and FVC) was electronically conducted by an investigator that did not take part in the evaluation of patients of application of the tests. Electronic codes were generated to introduce in the tDCS neuromodulator ensuring the blinding of the therapist and the subject assessed (doble blind). The volunteers of this research will be randomized in four groups: 20 volunteers per group, being 20 for young interventional and young placebo, and the same for the elderly. After a basal (day 1) evaluation all subjects will be part of two evaluation/intervention rounds with 48 to 72 h interval (Fig. 1).

### Subject data collection

Informed consent was obtained from every subject enrolled in the study.

A pre-test evaluation was performed for all volunteers in the study for data collection in electronic forms, collecting the following data:

- Demographic data: age, area of residence, social status, level of education.
- Anthropometric data and previous medical history: height, weight, BMI, respiratory pattern, blood pressure, peripheral saturation, chronic medication, previous medical conditions (particularly neurologic, respiratory and cardiovascular conditions), smoking status.
- IPAC-breve questionnaire was used to evaluate Physical Activity Index.

### Primary outcome measurements

Primary outcome is the increase in strength and lung function in the test groups compared to the sham group.

Lung function was performed with spirometry evaluation. Forced vital capacity (FVC), Forced Expiratory Volume in 1 S (FEV1), FEV1/FVC Ratio, Maximal Voluntary Ventilation (MVV) were the spirometry parameters evaluated using the SPM-A spirometer (CONTEC, China).

Inspiratory muscle endurance will be measured with Powerbreathe K5 equipment (Powerbreathe, Southam, England), using the S-INDEX, Flow and Volume. The handgrip strength will be checked using a hydraulic dynamometer (Saehan, Beijing, China).

### tDCS placement and technical aspects

**Fig. 2:**
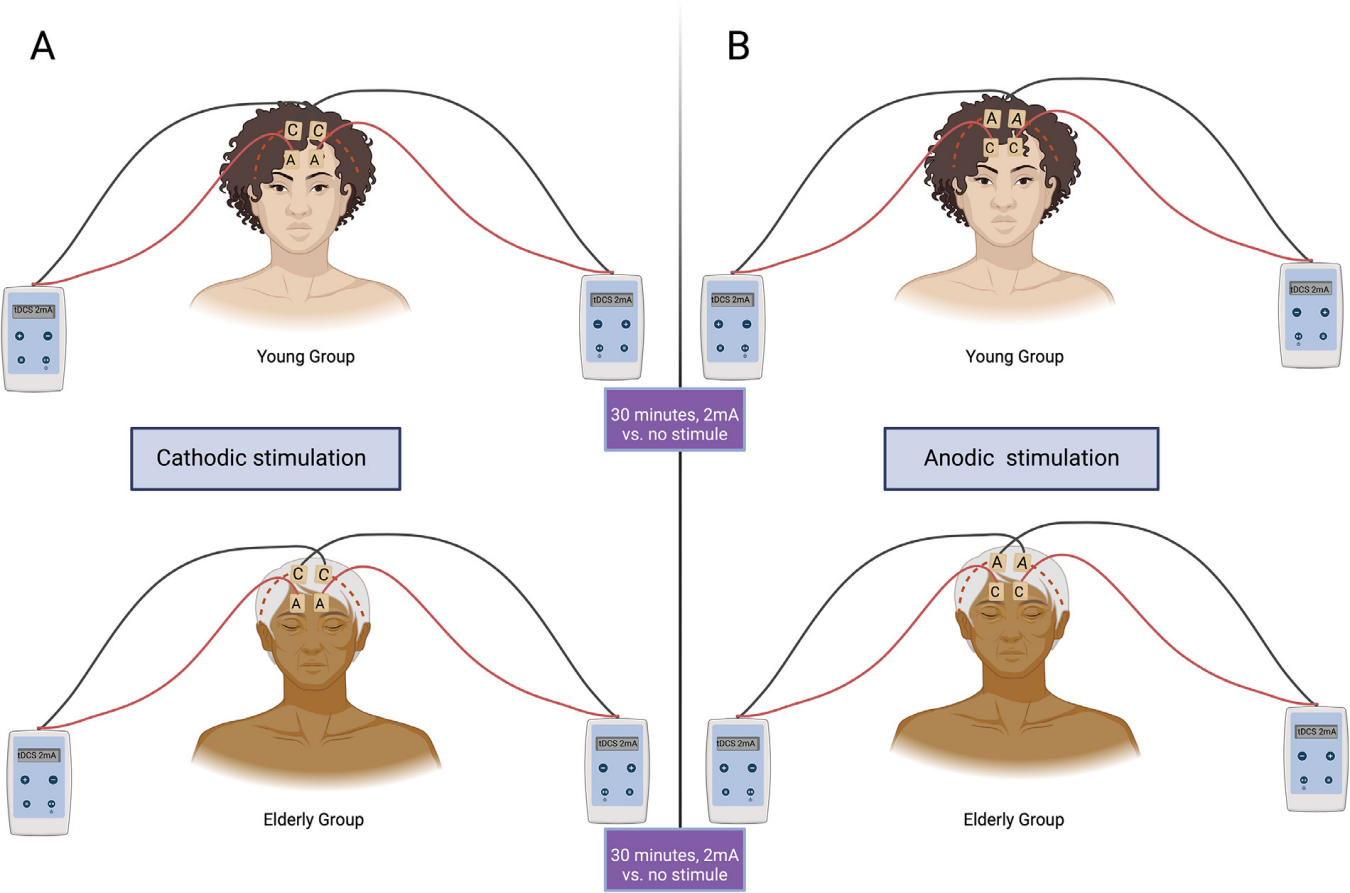
tDCS placement, A) cathodic stimulation; B) anodic stimulation. Both young and elderly group with a 30-minute 2 mA stimulation versus no stimulation.

For the intervention with the tDCS we used the neuromodulator from Microestim Foco Research, (NKL, Santa Catarina, Brazil). For every subject two devices were used to achieve bilateral stimulation. The cathode was placed in the C3 and C4 motor regions by 10/20 EEG bilaterally and the anode over the supraorbital region, also bilaterally – Fig. 2. All experiments were conducted with a 2 mA current for 30-minute period. The sham group will be subjected to the same protocol placement applied for intervention group; however, those volunteers will not receive any electrical stimulation, according to sham program from the device

The technician performing the intervention had previous experience in the use of tDCS in research and healthcare dataset.

### Step-by-step study design

The study is designed as an experimental and double blind randomized, summarized in Fig. 1.

### Day 1

All volunteers were evaluated initially for the presence of any exclusion criteria. After, a social demographic and anthropometric evaluation was conducted. Also, the patient's relevant medical history was recorded.

All subjects underwent baseline spirometry under controlled environmental conditions. The patient was seated and instructed to perform normal ventilations for 5 s, after a therapist command a slow expiration, followed by inspiration and forced expiration. For all participants, the device estimated a value of FVC and FEV_1_ by predicted values. A minimum of 3 and a maximum of 8 maneuvers were performed. At least 3 of these maneuvers would have to be acceptable and reproducible. Thus, differences greater than 10 % in the highest values of CVF or FEV_1_ were not admitted. The highest value of the series was chosen for both parameters in the study. The test would be stopped or restarted in the event of adverse effects such as cough or lipothymia.

### Day 2

All subjects were evaluated, within 24 h from day 1 evaluation, prior to any intervention, in quiet and temperature regulated conditions, with:

- Handgrip strength. The volunteers were seated with their feet well supported, with the elbow at 90° stabilized by the evaluator at the time of the measurement. The test was performed for 3 s, followed by a 1-minute break, with repetition of the intervention for two more rounds. The highest values of the 3 rounds were picked for the study.
- Spirometry evaluation with evaluation of the MVV. The patient was seated and was instructed to inhale and exhale quickly and deeply for 12 s. Up to 5 measurements were taken, choosing the one with the highest peak, as long as the variation was not greater than 10 %.
- Evaluation of dynamic inspiratory strength with Powerbreathe (Powerbreathe, Southam, England). With the subject seated and using a nose clip, the patient is instructed to inhale quickly and deeply through the mouthpiece with slow exhalation to the limit achieved. Followed by pause and repetition of the movement, The volunteers performed a warm-up of 5 inhalations before the assessment, which went up to a maximum of ten repetitions (Silva et al., 2018). The chosen value would be the maximum among the repetitions.

#### Intervention with tDCS

- The tDCS was performed in the test group with a 2 mA current for a 30-minute period, with the electrodes positioned has previously explained.
- During the intervention the patient was asked to remain quiet and still. The evaluator actively asked for the presence of any side-effect.

#### Post – intervention evaluation

- After the intervention with tDCS the patients repeated all the tests performed prior to intervention in the same technical and environmental conditions.

### Day 3

The evaluation in day 2 was conducted 48 to 72 h after the day 2 assessment. The environmental conditions were similar.

The pre and post intervention test were the same and conducted as described.

Regarding the intervention with tDCS the electrodes switched position with the anode being placed at C3 and C4 motor regions bilaterally and the cathode over the supraorbital region, also bilaterally. The intervention was then performed with the same parameters described for day 2 evaluation.

### Statistical analysis

The data will be expressed as mean and standard deviation of the mean, median and interquartiles, and percentage. Initially, normality will be tested using the Shapiro Wilk test and homogeneity using Levene's test. For mean analysis, the unpaired *t*-test or Mann-Whitney test will be used. For the evaluation of the intervention, ANOVA two-way (between-within) *post hoc* Bonferroni test will be used. If normal values are not present, the Kruskal-Walli's test will be performed for unpaired values and the Friedman test for paired data, with Bonferroni correction for the *p*-value. Effect size calculation will be done using the G*Power 3.1 software, and statistical analysis will be conducted using the JAMOVI software (Jamovi project, Australia) and GraphPad Prism 8.0 (GraphPad, USA). Significant values accepted as *p* < 0.05

## Protocol validation

Data analysis for the protocol is currently ongoing. An example of a JAMOVI data file for the analysis can be found in the Supplemental section. Data analysis includes ANOVA two-way (between-within) models.

## Limitations

Due to the scarcity of studies assessing the impact of respiratory motor stimulation with tDCS, conducting a clinical study without cross-sectionally testing the effect of transversal stimulation diminishes the impact of the study's blind and randomized clinical trial method. Moreover, this study will not combine inspiratory muscle training and tDCS, but again, it is necessary to conduct physiological studies to understand the behavior to increase the combination. Additionally, the lack of stimulation with TMS prevents the assessment of diaphragmatic muscle excitability. However, evaluating strength and endurance may pave the way for more complex studies with neuronavigation and the possibility of assessment in volunteers with cardiopulmonary diseases. In addition, the use of an all-female population may limit the robustness of the results, considering the noticeable physiological differences in lung capacity between men and women.

## CRediT author statement

**Elder Nascimento Pereira:** Conceptualization, Methodology, Writing – original draft preparation; **Elizângela Kuster, Márcia de Lorena Serra Maia, Jessica Oliveira da Silva, Laila Ramires Norbim:** Writing, Reviewing and Editing **Fernando Zanela da Silva Arêas, Jorge Henriques and Guilherme Peixoto Tinoco Arêas:** Supervision. Reviewing and Editing.

## Declaration of competing interest

The authors declare that they have no known competing financial interests or personal relationships that could have appeared to influence the work reported in this paper.

## Data Availability

Data will be made available on request.

## Appendix Supplementary materials

Image, application 1
