# Supplementary material for: Influence of bilateral transcranial direct-current stimulation on muscle strength and respiratory endurance: Randomized, placebo-controlled, double-blind trial protocol
Source: MethodsX. 2024 Sep 18;13:102939. doi: 10.1016/j.mex.2024.102939 (PMC11470191; doi:10.1016/j.mex.2024.102939)
Supplement: Supplementary file 1 [file mmc1.pdf]

Variáveis | Dados | Análises | Editar

Colar | Clipboard | Editar | Configurar | Calcular | Transformar | Adicionar | Apagar | Variáveis | Filtros | Adicionar | Apagar | Linhas

	Grupo	MMV pré	MMV pós	Sindex pre	Sindex pós	PFI pré	PIF pós	Volume p...	Volume p...	MMV pre...	MMV pre...
1	1	42.30	46.70	78.90	78.30	4.55	4.60	1.88	1.06	65.41	72.25
2	1	35.40	46.00	46.70	51.80	2.31	3.50	0.30	0.39	44.11	62.00
3	1	43.20	56.10	76.90	66.70	4.45	3.72	1.99	1.39	56.64	73.49
4	1	57.30	64.80	81.20	92.30	4.35	4.97	0.69	1.02	72.36	81.43
5	1	63.50	61.40	69.90	92.20	3.77	4.20	0.71	0.89	90.83	87.80
6	1	60.70	53.60	94.40	96.10	4.91	4.92	1.64	1.60	68.00	71.00
7	1	35.80	40.20	57.20	61.00	3.25	3.22	1.66	1.60	40.00	66.00
8	1	44.50	39.70	66.80	85.60	3.88	4.53	1.82	1.13	55.39	76.00
9	1	49.06	49.10	55.20	62.40	3.50	3.50	1.10	1.10	72.00	72.12
10	1	47.30	65.20	91.00	98.20	5.00	5.30	1.81	1.40	58.40	79.60
11	1	56.80	60.80	103.90	100.00	4.90	5.80	0.70	1.20	68.10	73.00
12	1	56.90	59.90	101.00	127.00	5.50	6.93	2.12	2.63	69.30	72.97
13	1	51.60	51.80	94.80	87.80	4.70	4.76	1.32	1.57	65.75	65.93
14	1	59.00	56.40	88.50	105.80	4.97	5.72	2.93	1.98	82.45	78.58
15	1	64.20	69.30	61.40	101.80	3.20	5.10	0.80	1.10	85.19	79.00
16	1	62.80	55.00	91.60	98.60	5.20	5.40	1.33	1.48	69.50	68.00
17	1	49.00	48.20	71.40	83.70	4.15	4.80	1.60	1.70	55.70	57.00
18	1	44.71	43.73	60.43	92.22	3.50	4.98	1.80	0.78	57.49	56.20
19	1	45.61	50.92	71.37	68.24	3.56	3.45	0.85	0.50	62.22	69.47
20	2	51.30	60.20	74.30	85.50	4.31	4.75	1.67	1.67	57.93	67.97
21	2	49.20	49.80	71.50	77.00	3.81	3.81	1.57	1.57	74.82	75.70
22	2	52.90	53.90	94.70	98.90	5.25	5.59	2.14	2.14	58.13	59.26
23	2	54.30	77.20	74.60	78.20	4.29	4.31	1.84	1.22	68.54	77.19
24	2	55.20	35.50	67.70	65.10	3.93	3.78	1.12	1.44	71.18	45.80
25	2	45.70	55.80	81.00	85.30	4.43	4.73	0.77	0.98	54.50	66.58
26	2	39.80	41.30	71.80	76.20	4.07	4.41	0.08	1.61	50.28	52.08
27	2	47.80	52.20	81.50	89.20	4.69	4.77	1.53	1.72	60.73	66.81
28	2	48.80	45.50	74.03	83.84	4.10	4.38	0.75	1.61	61.49	57.25
29	2	55.40	59.90	76.60	92.72	4.43	5.00	1.40	1.20	73.08	79.05
30	2	54.30	54.40	110.85	101.47	5.70	5.60	1.42	1.50	67.42	67.62
31	2	69.50	66.10	102.00	80.40	5.60	4.50	1.99	1.19	89.74	85.43
32	2	36.90	41.60	58.54	72.96	3.30	4.70	1.07	1.21	49.95	56.30
33	2	50.50	60.00	57.00	78.50	3.20	4.42	1.34	1.31	70.64	83.84
34	2	50.70	43.20	62.60	77.20	3.50	3.97	0.78	0.59	63.40	55.80
35	2	63.50	61.50	88.70	85.20	5.05	4.80	1.50	1.73	84.00	79.00
36	2	42.70	56.80	76.90	78.30	4.20	4.30	1.07	1.02	58.80	78.20

Pronto | Filtros 0 | Contagem em linha 40 | Filtr
